# The Prevalence of Alveolar Ridge Defects According to Seibert's Classification: A Cross-Sectional Study

**DOI:** 10.7759/cureus.75602

**Published:** 2024-12-12

**Authors:** Mohamed Farith N, Kalyani Ramkumar Sadhana, Vidyashree V Nandini

**Affiliations:** 1 Prosthodontics, SRM Kattankulathur Dental College and Hospital, SRM Institute of Science and Technology, Chengalpattu, IND

**Keywords:** alveolar ridge defect, bone loss, partially edentulous, prevalence rate, prosthodontics related

## Abstract

Background and objective

Alveolar ridge defects in partially edentulous patients present significant challenges in prosthodontic treatment planning. Seibert's classification system provides a structured approach to categorizing these defects based on the buccolingual and apico-coronal dimensions of the ridge. Accurate classification is crucial for determining appropriate treatment strategies for implant placement, fixed prosthesis, or tissue augmentation. Hence, this study aimed to assess and classify tissue defects in partially edentulous ridges based on Seibert's classification

Methods

A cross-sectional study was conducted to measure and classify alveolar ridge defects in partially edentulous patients by using Seibert's classification system. Patients aged 18 years and above with partially edentulous ridges requiring prosthodontic treatment were considered for inclusion. Individuals with systemic conditions affecting healing, completely edentulous patients, and those with active periodontal infections were excluded. Impressions of dental arches were obtained, and the alveolar ridge dimensions were measured using digital calipers.

Results

The study included 122 participants, with a slight male predominance (n=65, 53.3%). The classification of alveolar ridge defects revealed that 71 (58.2%) were Class III, 33 (27.0%) were Class I, and 18 (14.8%) were Class II. A chi-square analysis indicated no significant association between sex and the classification of alveolar ridge defects (p=0.410).

Conclusions

The prevalence of Class III alveolar ridge defects highlights the need for tailored treatment approaches in prosthetic rehabilitation. While gender differences were observed, they did not significantly influence defect classification. Future research should investigate additional demographic factors to enhance understanding and improve treatment strategies.

## Introduction

Treating patients with missing teeth and bone loss in the jaw can be challenging for prosthodontists. Alveolar ridge defects, a common consequence of tooth loss, pose a significant challenge in prosthetic rehabilitation. Local alveolar ridge defects are characterized by a limited area of missing bone and soft tissue within the alveolar process. This can occur due to tooth loss, traumatic extractions, or congenital issues. The loss of bone leads to the overlying soft tissue sinking inward during healing, resulting in an altered ridge shape [1-3].

To achieve better treatment planning, clinical outcomes, and prognoses, it is crucial to consider the type and extent of damage to the residual ridge and the patient's overall health. Choosing the right pontic is essential and may even necessitate surgical intervention to reshape the ridge. These classifications categorize ridge defects based on quantity and quality, with Seibert's classification being the most prevalent and widely used [4-8].

These defects can vary in severity and location, impacting treatment success. Seibert's classification system provides a comprehensive framework for understanding and categorizing these defects. It distinguishes three types of alveolar ridge defects based on the location of bone loss: 

Class 1: Characterized by bone loss affecting the buccal and lingual aspects of the edentulous area of the ridge. 

Class 2: Defined by bone loss affecting the occlusal and cervical aspects of the edentulous area of the ridge. 

Class 3: A combination of both Class 1 and Class 2 bone loss, representing the most complex scenario.

The treatment approach varies for each Seibert's class. Understanding the prevalence and classification of alveolar ridge deformities is crucial for guiding treatment strategies and optimizing outcomes in prosthetic rehabilitation. Based on Seibert's classification, this study employed a cross-sectional design to measure and classify tissue defects in partially edentulous ridges [9].

## Materials and methods

Study design and setting

The cross-sectional study involved 122 partially edentulous male and female patients at the Department of Prosthodontics, SRM Kattankulathur Dental College & Hospital, Tamil Nadu. The sample size was calculated using G*Power software for mean differences. At a 5% level of significance and 95% power with a standard effect size of 0.3, the total sample size was determined to be 122 [10,11].

Inclusion and exclusion criteria

Patients aged 18 years and above, individuals with partially edentulous ridges requiring prosthodontic treatment with a minimum healing period of three months post-extraction (to ensure ridge stabilization), and patients willing to participate in the study were included. Patients with systemic conditions such as diabetes mellitus, osteoporosis, autoimmune disorders, vitamin D deficiency, immunosuppressive disorders, and other relevant conditions affecting bone and tissue healing, complete edentulous patients, and those with active periodontal infections that could interfere with the measurements were excluded [12].

Methodology

**Figure 1:**
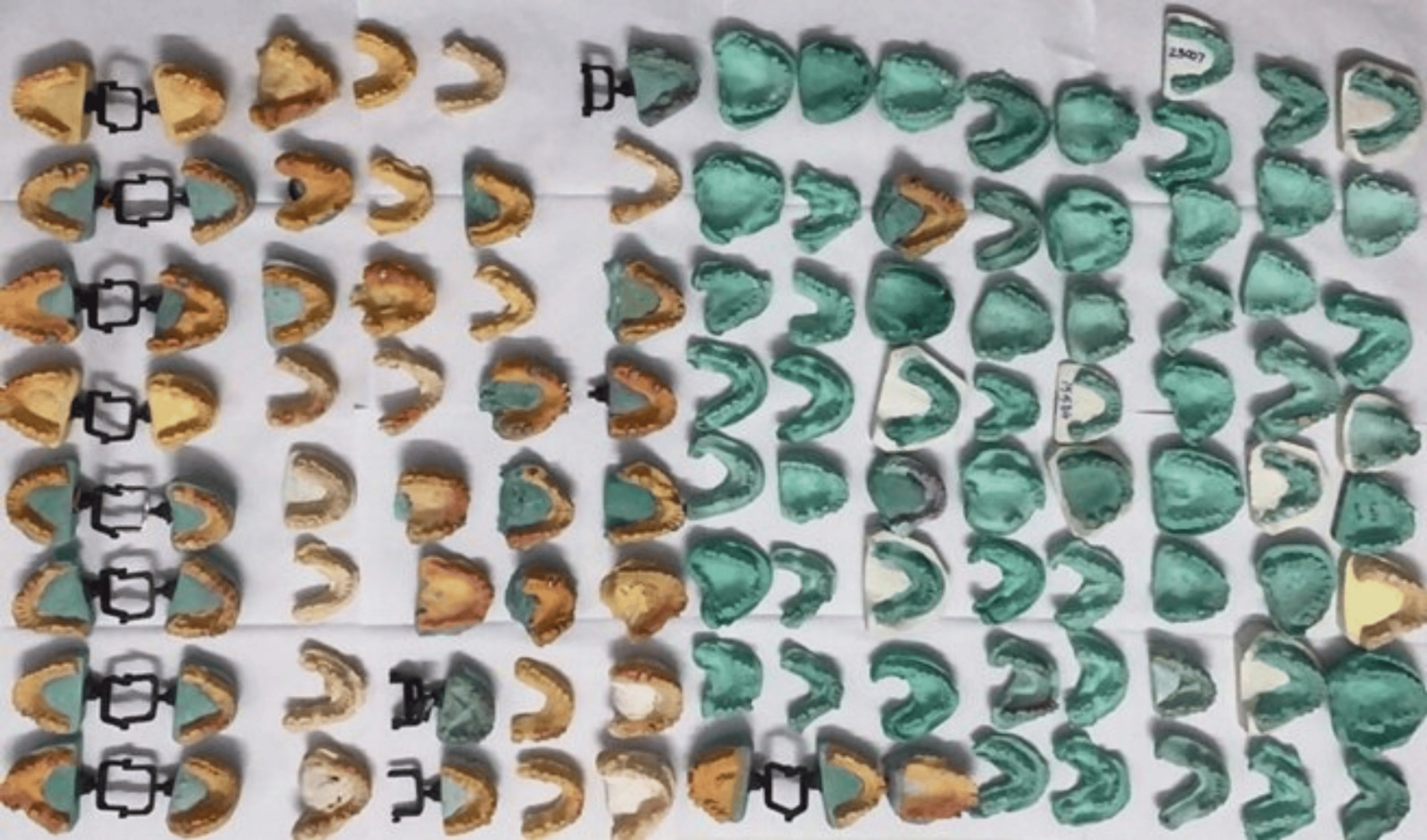
Representative photograph of diagnostic casts used in this study

Diagnostic impressions of partially edentulous dental arches were made using alginate (irreversible hydrocolloid) material and a single-stage technique for patients with the chief complaint of missing teeth and seeking replacement with prosthetic teeth. Diagnostic casts were fabricated; the casts were subjected to measurement of the buccolingual and apico-coronal dimensions of the partially edentulous ridges at the crestal level of the residual ridge, and the defects were classified based on Seibert’s classification and documented accordingly. These casts are crucial for evaluating the alveolar ridge dimensions and classifying the defects on Seibert’s classification (Figure 1). The measured dimensions were used to classify the alveolar ridge defects according to Seibert's classification [13,14].

Data and statistical analysis

Data analysis was performed using SPSS Statistics software, version 28.0 (IBM, Armonk, NY). Descriptive statistics were applied, with categorical variables presented as frequencies and percentages. Statistical associations were evaluated using the chi-square test. A p-value of less than 0.05 was considered statistically significant [15].

Ethical considerations

The Institutional Ethics Committee, SRM Medical College Hospital and Research Centre granted the ethical clearance (ref. no: SRMIEC-ST0924-1731). All participants provided informed consent before data collection. Data were handled confidentially and anonymized to protect participants’ privacy. The study adhered to the institution's ethical guidelines [16,17].

## Results

This study population comprised 122 partially edentulous patients with alveolar ridge defects, of whom 65 (53.3%) were male and 57 (46.7%) were females (Table 1). Understanding these demographic characteristics is crucial, as gender could influence the study's outcomes, particularly in terms of how alveolar ridge defects are manifested or reported [18-21].

**Table 1:** Distribution of participants by gender

	Frequency	Percentage	Cumulative percentage
Male	65	53.3	53.3
Female	57	46.7	100.0
Total	122	100.0	

According to Seibert’s classification, the most common type of alveolar ridge destruction was the Class III defect, which involves both buccolingual and apico-coronal tissue loss. This accounted for 71 cases (58.2%). The second most prevalent was the Class I defect, characterized by buccolingual tissue loss (n=33, 27%). The least common was the Class II defect, which involves apico-coronal tissue loss, affecting 18 patients (14.8%) (Table 2).

**Table 2:** Frequency of defects based on Seibert's classification Class I: Only buccolingual tissue loss. Class II: Only apico-coronal tissue loss. Class III: Both buccolingual and apico-coronal tissue loss

	Frequency	Percentage	Cumulative percentage
Class I	33	27.0	27.0
Class II	18	14.8	41.8
Class III	71	58.2	100.0
Total	122	100.0	

Crosstabulation provided the distribution of alveolar ridge defects according to Seibert's classification, with the data categorized by sex. This breakdown provides a clear comparison of the distribution of each defect type (Class I, Class II, and Class III) between male and female participants (Table 3) [22-24].

**Table 3:** Crosstabulation between gender and Seibert's classification of alveolar ridge defects

			Seibert's classification class			Total
			Class I	Class II	Class III	
Gender	Male	Count	18	7	40	65
		% of total	14.8%	5.7%	32.8%	53.3%
	Female	Count	15	11	31	57
		% of total	12.3%	9.0%	25.4%	46.7%
Total		Count	33	18	71	122
		% of total	27.0%	14.8%	58.2%	100.0%

Chi-square test

The Pearson chi-square and likelihood ratio tests revealed no significant association between gender and Seibert's classification of alveolar ridge defects, with p-values of 0.410 and 0.409, respectively (Table 4). Based on 122 valid cases, the results suggested that gender does not influence the distribution of these defects [25].

**Table 4:** Chi-square test results ^a^The minimum expected count is 8.41, and no cells (0%) have an expected count of less than 5

	Value	df	Asymptotic significance (2-sided)
Pearson chi-square	1.786^a^	2	0.410
Likelihood ratio	1.788	2	0.409
Linear-by-linear association	0.133	1	0.715
No. of valid cases	122		

## Discussion

The treatment approach and prognosis for patients with alveolar ridge defects are closely linked to the severity of the existing condition. This study, which utilized Seibert’s classification system, found that Class III defects involving buccolingual and apico-coronal tissue loss were the most common, affecting 58.2% of patients. This was followed by Class I defects (27%), characterized by buccolingual tissue loss, and Class II defects (14.8%), defined by apico-coronal tissue loss.

When examining the distribution of these defects by gender, some interesting trends emerged. Class III defects were the most prevalent in male (61.5%) and female (54.4%) participants, although males had a slightly higher incidence. Class I defects showed a similar distribution across both genders, with 27.7% of males and 26.3% of females affected. In contrast, Class II defects were more common in females (19.3%) than males (10.8%). Also, while there were some gender-related differences in the distribution of Class I and Class II defects, Class III defects remained the most frequent type of alveolar ridge destruction in both males and females. These findings highlight the importance of considering both the severity of the defect and gender when developing treatment plans and predicting outcomes for patients with alveolar ridge defects. Understanding these patterns can help clinicians tailor their approach to each patient’s specific condition, thereby improving the chances for successful treatment outcomes [26-28].

The objective of studying the prevalence of alveolar ridge defects using Seibert's classification was to provide a clearer understanding of treatment options and strategies for achieving successful outcomes. The primary goal in treating these defects is to restore function, aesthetics, and natural appearance by closing the defect and replacing the missing tooth. However, this can only be accomplished if the final prosthesis is tailored to the specific defect pattern. The prevalence of alveolar ridge defects in our study closely mirrors that of previous research. Abrams et al. have reported that 91% of partially edentulous patients had anterior ridge deformities, almost identical to the 91.6% prevalence in our study [5]. Similar to our findings, they also noted a predominance of Class III defects, accounting for 55.8%, followed by Class I (32.8%), and Class II defects (2.9%). Further supporting these trends, John et al. highlighted that posterior mandibular bone defects were the most prevalent, with Class III defects being the most frequent overall. Given that Class III defects are the most common, several studies and case reports have focused on the treatment outcomes for these defects, emphasizing their clinical significance and the need for customized treatment plans [9,29].

Limitations

This study has a few limitations. The cross-sectional design limited the ability to track changes in defects over time. Furthermore, the study did not consider possible confounding factors, such as patient demographics or medical history, which may have impacted the results. Lastly, focusing on a specific population may affect the broader applicability of the findings [30].

## Conclusions

This study highlights the complexity of managing alveolar ridge defects in partially edentulous patients. It indicates that while Class III defects are more prevalent, their distribution is not significantly influenced by gender. The prevalence of Class III defects further emphasizes the need for individualized, defect-specific approaches in prosthetic rehabilitation to optimize outcomes. For clinicians, recognizing these patterns is essential for personalizing diagnostic and treatment plans. Future research should explore additional variables, such as age and other demographic factors, including different populations, to clarify their potential influence on alveolar ridge classifications.
